# Is intergenerational care associated with depression in older adults?

**DOI:** 10.3389/fpubh.2024.1325049

**Published:** 2024-01-29

**Authors:** Shurong Han, Jiahui Guo, Jianjun Xiang

**Affiliations:** ^1^International Business School, Shaanxi Normal University, Xi’an, China; ^2^Department of Preventive Medicine, School of Public Health, Fujian Medical University, Fuzhou, China

**Keywords:** intergenerational care, depression, older people, propensity score matching, heterogeneity

## Abstract

**Background:**

It has become an alarming issue that older adults in China are facing mental health issues, causing severe depression. In this context, intergenerational care, in which grandparents care for young children instead of the young parents, is gaining importance. This study aims to explore the correlation between intergenerational care and depression among older adults, seeking alternative approaches to enhancing their quality of life. This study concludes that intergenerational care is an effective strategy for promoting active aging.

**Methods:**

This study used multiple linear regression, propensity score matching, and additional analysis of heterogeneity, mediation, and moderation effects, with data from the 2018 CHARLS survey.

**Results:**

The results indicated a negative correlation between intergenerational care and depression among older adults. The correlation was stronger for females and younger older adults people. Additionally, social activities served as a mediator between intergenerational care and depression among older adults, and health satisfaction positively moderated this relationship.

**Conclusion:**

This study posits that intergenerational care serves as an effective approach for promoting active aging. It emphasizes the necessity for supportive government policies and community-family collaborations to encourage intergenerational care and its beneficial impact on mental health among older adults.

## 1 Introduction

By the year 2021, the population of Chinese citizens aged 60 or over had reached 267 million, making up a significant 18.9% of the total population. Based on the 2019 World Population Prospects Report by the United Nations Population Division, it is anticipated that China’s population aging rate will increase to 20.7% in 2035, and 26.1% in 2050 (1). This growing population of older adults people has caused heightened concerns about their health. As people age, their physical abilities deteriorate, and they must deal with new challenges associated with changing social roles and lifestyle changes. These changes can have a significant negative impact on their mental well-being, leading to feelings of anxiety, depression, and loneliness. In a survey conducted by the National Health and Wellness Commission, it was discovered that the prevalence of mental health issues among older adults in urban areas was 30.3%, while in rural areas, it was slightly lower at 26.8% (2). This alarming statistic suggests that the mental health of older adults in China is a serious issue that requires further attention. Depression is a common and noticeable manifestation of poor mental health.

Traditionally, older people have been seen as a group that requires assistance. However, as their health improves and their life expectancy increases, this perception of being reliant on others for care is gradually fading away. A significant approach to this is intergenerational care, which is a family upbringing model where younger parents are unable to provide sufficient care for their underage children due to various reasons, and their grandparents step in to raise, nurture, and educate them (3). As a result of East Asian cultural norms, the challenges faced by single-child families in childcare, and changes in family dynamics due to the rise in female workforce participation, intergenerational care has become a widespread choice for families (4). Intergenerational care is deeply entwined with the lives of older people, and its impact on female workforce participation and the market’s childcare service demand–supply balance can potentially increase fertility rates. However, its influence on the mental health of older adults themselves requires further investigation. In 2002, the World Health Organization introduced the concept of “active aging,” a policy framework aimed at enhancing the quality of life and providing older people with opportunities for health, participation, and security in the face of population aging in the 21st century (5). Therefore, exploring the connection between intergenerational care and the mental health of older people is crucial for advancing their well-being and promoting “active aging.”

The relationship between intergenerational care and the mental health of older people is a complex and contentious issue. There are currently two competing perspectives on this matter. One view, endorsed by the role tension theory, argues that intergenerational care negatively affects the mental health of older individuals. This theory posits that individuals in society are assigned various roles, each with different demands and expectations. As individuals attempt to juggle these diverse roles, they can experience role conflict, which can manifest as stress and strain, ultimately having a detrimental impact on their mental health (6). The strain imposed on older individuals by intergenerational care is accentuated by the physical and emotional toll it demands. This care often requires substantial effort, leaving older individuals overwhelmed due to their physical limitations (7, 8). Furthermore, the stress and dysfunctionality that often accompany family caregiving can have a particularly detrimental effect on older individuals’ mental health (9). The routine and mechanical nature of caregiving can lead to feelings of loneliness and boredom, which are known to negatively impact mental health (10). In addition, older individuals often prioritize the care of their grandchildren, thereby neglecting their own mental health, leading to a decline in their overall health (11). The psychological impacts of caregiving for older individuals can include depression and familial pressures (12). A study in the United States found that African American grandparent caregivers experience more depressive symptoms compared to their noncaregiving peers (13). Despite this, the relationship between the intensity of intergenerational care and the mental health of older individuals remains fluid. High-intensity caregiving can crowd out older individuals’ time and energy for self-care, negatively affecting both their physical and mental health (14, 15). On the other hand, some scholars suggest that high-intensity intergenerational care can reduce feelings of loneliness in older individuals, while low-intensity care can negatively affect their self-rated health (16).

The mental health of older individuals can be positively influenced by intergenerational care, which is supported by the role enhancement theory (17). This theory was developed by Sieber in 1974, building on the role tension theory. It posits that taking on multiple roles can not only cause role tension but also lead to benefits such as material resources and spiritual well-being. Intergenerational care can enable older individuals to increase their material and spiritual resources by assuming caregiving responsibilities. This practice can alleviate family stress associated with child care (18) and provides older individuals with more intergenerational support from their children (19). Moreover, providing intergenerational care allows older individuals to demonstrate their worth (20), find enjoyment and enrichment in their later years (21, 22), strengthen their family relationships (23), and enhance the bond between grandparents and grandchildren (24, 25). Zhao, J. found that reverse feeding, a form of intergenerational care where parents care for their children, can improve older individuals’ mental health (26). Furthermore, older individuals who assist their children with daily tasks can boost their abilities and sense of authority, which in turn can enhance their subjective well-being (27). The mental health of older individuals is significantly dependent on family companionship (28), and traditional Chinese culture advocates for older individuals to maintain productivity through caring for their grandchildren or household chores (29).

Cultural diversity across countries and ethnicities contributes to different motivations for older adults to engage in intergenerational care. Catherine and Merril examined the experiences of 1,058 grandparents with diverse ethnic backgrounds who had cared for their grandchildren in Los Angeles, United States. They discovered that the varying cultural backgrounds and lifestyles of these grandparents resulted in differences in their willingness to provide intergenerational care. This directly impacted the relationship between intergenerational care and their health status (30).

In China, a cultural context that values family and inheritance, older individuals tend to be more willing to take on intergenerational care as a way of perpetuating the family legacy (31). This sense of obligation in China often leads to a higher voluntary participation in intergenerational care among grandparents, with 51.7% of middle-aged and older Chinese individuals caring for their grandchildren (32). This compared to only 5.9% of middle-aged and older Korean individuals providing care to their grandchildren (33). This demonstrates that the act of providing intergenerational care is viewed more as a duty in China, while in Korea it is viewed as an optional act.

Moreover, there are multiple factors that influence the relationship between intergenerational care and older individuals’ depressive status, including gender, age, mobility, cognitive appraisal, time of care initiation, and source of care recipient (34–37). The gender division of labor theory suggests that men are typically associated with the public sphere and engaged in productive work, while women are associated with the private sphere and engaged in work related to human reproduction (38). Even in old age, men continue to be less involved in family matters. Wu Qi analyzed 180 questionnaires in Nantong, Jiangsu province, China, and found that the primary reason for rural older individuals to move to the city was to care for their grandchildren (39). Among the older individuals surveyed, women accounted for 80, and 90% of them were willing to take on the primary responsibility of caring for their grandchildren. The level of intergenerational care varies among older individuals of different ages. Younger older adults individuals are more actively involved in intergenerational care, whereas older individuals may no longer have grandchildren in need of care (40), and their physical condition as they age may also make them unable to provide intergenerational care. Therefore, intergenerational care varies based on gender and age, leading to differences in depressive status.

The mental health of older individuals is positively linked to social support (41), as suggested by the social companionship model, which posits that social support is derived from a person’s social network, which may vary throughout life depending on their circumstances (42). The presence of a supportive social network is vital for the physical and mental health of individuals, especially older adults, as it reduces stress and the likelihood of disabilities. The positive impact of social networks on the mental health of older adults has been extensively studied (43, 44), and it has been observed that individuals with fewer social connections are more susceptible to depression (45). Social activities, an integral part of social support, have been proposed as a way to increase the frequency and likelihood of social engagement in older adults, potentially impacting their mental state.

Previous research has highlighted that the impact of intergenerational care on health outcomes is largely contingent upon the preferences and satisfaction of the caregiver (46). This satisfaction, in turn, represents an overall evaluation of both physical and mental well-being. Thus, differences in self-evaluations of health can influence the perceived benefits of intergenerational care, either enhancing activities and cognitive functions (47), or potentially causing discomfort and role overload (48). These disparities in health satisfaction may subsequently alter older adults’ subjective perceptions of intergenerational care and subsequently influence its association with mental health.

Despite these significant insights, there are several limitations to consider. In particular, many studies have not taken into account selection bias, an issue that causes endogeneity. Therefore, in this study, we used propensity score matching to address this concern. Additionally, while caregivers may exhibit individual differences, there has been relatively little research examining gender and age disparities in intergenerational care. Therefore, our research will aim to fill this gap. Furthermore, previous studies have not thoroughly explored the intermediate mechanisms by which intergenerational care influences mental health. Hence, our study will aim to investigate the potential impact of social activities and health satisfaction on the relationship between intergenerational care and depression. Guided by these considerations, we propose the following hypotheses:

*H1*: Depression among older adults is negatively associated with intergenerational care.

*H2*: The relationship between intergenerational care and depression varies among older adults of different genders and ages.

*H3*: Social activities mediate the relationship between intergenerational care and depression among older adults.

*H4*: Satisfaction with one's own health positively moderates the relationship between intergenerational care and depression among older adults.

## 2 Materials and methods

The data used in this study were collected by the National Development Research Institute of Peking University and the China Social Science Survey Center through the China Health and Retirement Longitudinal Survey (CHARLS). The survey collects data in four waves, spanning from 2011 to 2018. These data cover approximately 150 county-level units, 450 village-level units, and 10,000 households. In our study, we used the latest data from 2018. CHARLS makes its data publicly available to the academic community 1 year after collection, meaning that the data we used were not collected by us and no ethical approval was required. The survey provides comprehensive information on demographics, households, health status and functioning, cognition and depression, healthcare and insurance, and pensions.

The main focus of this study was the analysis of a sample of individuals aged 60 years and above, specifically focusing on older adults, a population defined as individuals over the age of 60 according to the Law on the Protection of the Rights and Interests of older adults. Once the relevant observations with missing data were excluded, the final sample was comprised of 3,701 female and 3,890 male older adults.

The dependent variable for this study was the mental health of older adults, specifically depression. This was measured using a questionnaire, which asked 10 questions about depression, each offering four possible responses: “Rarely or not at all (<1 day),” “Not very much (1–2 days),” “Sometimes or about half the time (3–4 days),” and “Most of the time (5–7 days).” The questions “I am hopeful about the future” and “I am happy” were reverse-coded, with scores of 3, 2, 1, and 0 assigned, while the remaining eight questions were positively scored, with scores of 0, 1, 2, and 3 assigned. The scores of these 10 questions were then summed to create a score indicating the level of depression among older adults, which ranged from 0 to 30.

The intergenerational care variable was binary, indicating whether or not respondents had spent time caring for their grandchildren in the past year. The survey asked if the participants had spent time taking care of their grandchildren in the past year, and this was selected for 3,131 caregivers. The survey also asked for the specific time when they provided intergenerational care last year. This article calculated the total time of intergenerational care by adding up the time spent caring for different grandchildren. Therefore, if an older adults person cares for multiple grandchildren, the intergenerational care time is the total amount of time provided for multiple periods of intergenerational care. The intergenerational care time was measured as a continuous variable, ranging from 0.5 weeks to 208 weeks. The smaller the value, the shorter the time spent providing intergenerational care.

For the intermediate variable, social activity, we determined the participant’s level of social activity by looking at their answer to the CHARLS question: “Have you done any of the following social activities in the past month (multiple choices possible)?” If they participated in one or more of the 11 social activities, we assigned them a value of “1 = social activities”; if they did not participate in any of the activities, we assigned them a value of “0 = no social activities.”

For the adjustment variable, health satisfaction, we measured how satisfied participants were with their health by looking at their answer to the CHARLS question: “How satisfied are you with your health?” We used a 3-point scale and defined “0 = unsatisfied” as not very satisfied or not at all satisfied, “1 = satisfied” as completely satisfied, very satisfied, or somewhat satisfied, and “2 = not sure” as unsure or neutral.

In addition to the intermediate and adjustment variables, we also considered other factors that could affect participants’ responses. These included demographic factors such as gender, age, education level, marital status, living arrangement, and financial support from father to son or from son to father, and relational factors such as satisfaction with the relationship with children. All of these factors were derived from the questionnaire’s corresponding question and answer items. For some of these factors, we combined variables based on previous studies to create new variables. Table 1 provides detailed definitions and assignments of these control variables.

**Table 1:** Variable descriptions.

Type	Variables	Variable definitions
Dependent variable	Depression	0–30, the lower the score, the less depressed you are
Independent variable	Intergenerational care	0 = no intergenerational care; 1 = with intergenerational care
	Intergenerational care time	0.5-208,the smaller the variable, the shorter the time to provide intergenerational care
Intermediate variable	Social activities	0 = no social activity; 1 = with social activity
Control variable	Gender	0 = female; 1 = male
	Age	60–108 years
	Education level	0 = no education; 1 = primary education; 2 = secondary education; 3 = tertiary education
	Marital status	0 = no spouse; 1 = with spouse
	Living arrangements	0 = not living with children; 1 = living with children
	Financial support from son to father	0 = no financial support; 1 = with financial support
	Financial support from father to son	0 = no financial support; 1 = with financial support
	Satisfaction with relationship with children	0 = unsatisfied;1 = satisfied
	Health satisfaction	0 = unsatisfied;1 = satisfied

## 3 Results

### 3.1 Descriptive statistics

Table 2 presents the descriptive statistics for the main variables, based on a sample size of 7,591. The sample consisted of 51.24% males, with a relatively balanced ratio of males to females. The average age was 68.2 years, with 83.44% of the sample aged between 60 and 75 years. The level of education was uneven, with the majority of older people having received only primary education or less and only 4.27% having received higher education. Among the older people, 82.56% had a spouse, with 66.59% not living with their children. The percentage of children providing financial support for their father was 63.39%, while the percentage of fathers providing financial support for their children was only 30.73%. The majority of older people were satisfied with their relationship with their children, with only 4.47% expressing not very satisfied or not at all satisfied. Older people showed some variation in their satisfaction with their health, with 73.48% expressing satisfaction. Participation in social activities was evenly distributed, with 50.78% of older people engaging in social activities. The mean score for depression among older people was 8.713. Intergenerational care was common in the sample, with 43.97% of older people providing intergenerational care for their grandchildren and 56.03% not providing intergenerational care.

**Table 2:** Descriptive statistics results.

Variables	Variable classification	Sample size	*M*	SD
Depression		7,591	8.713	6.698
Intergenerational care	No intergenerational care (56.03%)	7,591	0.440	0.496
	With intergenerational care (43.97%)			
Intergenerational care time		3,131	46.495	27.486
Social activities	No social activities (49.22%)	7,591	0.508	0.500
	With social activities (50.78%)			
Gender	Female (48.76%)	7,591	0.512	0.500
	Male (51.24%)			
Age	60–75 years (83.44%)	7,591	68.206	6.151
	Over 75 years old (16.56%)			
Education level	Not receiving education (25.71%)	7,591	1.063	0.812
	Primary education (46.52%)			
	Secondary education (23.50%)			
	Higher education (4.27%)			
Marital status	No spouse (17.44%)	7,591	0.826	0.379
	With spouse (82.56%)			
Living arrangements	Not living with children (66.59%)	7,591	0.334	0.472
	Living with children (33.41%)			
Financial support from son to father	No financial support (36.61%)	7,591	0.634	0.482
	With financial support (63.39%)			
Financial support from father to son	No financial support (69.27%)	7,591	0.307	0.461
	With financial support (30.73%)			
Satisfaction with relationship with children	Unsatisfied (4.47%)	7,591	0.955	0.207
	Satisfied (95.53%)			
Health satisfaction	Unsatisfied (26.52%)	7,591	0.735	0.441
	Satisfied (73.48%)			

Data source: China Health and Retirement Longitudinal Study Wave 4 (2018).

Additionally, this article divided the sample into two groups: those without intergenerational care and those with intergenerational care, and conducted intergroup difference tests (Table 3). The t-test was used to compare the mean differences between the two groups for depression and age, while chi-square tests were used for the rest of the variables. Significant intergroup differences were found for all variables except financial support from son to father and satisfaction with the relationship with children.

**Table 3:** Intergroup difference test.

Variables	Variable classification	No intergenerational care	With intergenerational care	Difference in means	Pearson chi-square (value)	*P*
Depression				0.75***		
Age				4.06***		
Social activities	No social activities	29.14%	20.08%		30.21	0.00
	With social activities	26.89%	23.90%			
Gender	Female	27.95%	20.80%		5.02	0.03
	Male	28.07%	23.17%			
Education level	Not receiving education	15.83%	9.88%		110.27	0.00
	Primary education	27.15%	17.37%			
	Secondary education	10.74%	12.77%			
	Higher education	2.31%	1.96%			
Marital status	No spouse	12.05%	5.39%		111.40	0.00
	With spouse	43.97%	38.59%			
Living arrangements	Not living with children	40.44%	26.15%		135.96	0.00
	Living with children	15.58%	17.82%			
Financial support from son to father	No financial support	20.25%	16.36%		0.92	0.34
	With financial support	35.78%	27.61%			
Financial support from father to son	No financial support	39.48%	29.79%		6.56	0.01
	With financial support	16.55%	14.19%			
Satisfaction with relationship with children	Unsatisfied	2.57%	1.90%		0.32	0.57
	Satisfied	53.46%	42.08%			
Health satisfaction	Unsatisfied	15.36%	11.16%		4.00	0.05
	Satisfied	40.67%	32.82%			

Data source: China Health and Retirement Longitudinal Study Wave 4 (2018). Standard errors in parentheses, *p < 0.1; **p < 0.05; ***p < 0.01; the *t*-test is used to test the intergroup differences between depression and age, while the rest are chi-square tests.

### 3.2 Regression analysis

A multiple linear regression model (OLS) was used to explore the correlation between intergenerational care and the depressive status of older people. In the analysis below, age was standardized. In Model 1, the relationship between a range of control variables and depression was examined. These included gender, age, education level, marital status, living arrangements, and financial support from children to their parents. The study found that male older people, those who were older, had a higher level of education, were married, lived with their children, received financial support from their children to their parents, and were satisfied with their relationship with their children had varying levels of depression. Furthermore, the study found that those who received no financial support from their children to their parents, received financial support from their parents to their children, or were satisfied with their relationship with their children had better mental health status. The study further found that older, better educated older people were more self-adjusted, more tolerant of life, and more capable of relieving tension and stress. Those who were married, lived with their children, or were satisfied with their relationship with their children had a higher level of family well-being and had more options to relieve negative emotions. Finally, those who could provide financial support for their children but did not need financial support from their children were more confident and free.

Model 2 of the study analyzed the correlation between intergenerational care and depression, with a regression coefficient of −0.333 (*p* < 0.05). Model 3 explored the relationship between intergenerational care time and depression, with a regression coefficient of 0.006 (*p* < 0.1). The study found that providing intergenerational care was associated with lower levels of depression, but that a moderate care time was needed to maintain positive mental health.

The analysis of the data in Table 4 supported the hypothesis that depression among older people is negatively related to intergenerational care, which supports the H1 hypothesis.

**Table 4:** Regression results of intergenerational care and intergenerational care time on depression of older people.

	(1)	(2)	(3)
Variable name	Depression	Depression	Depression
Intergenerational care(no = 0)		−0.333**	
		(−2.27)	
Intergenerational care time			0.006*
			(1.75)
Gender(female = 0)	−1.040***	−1.036***	−0.851***
	(−7.03)	(−7.00)	(−3.83)
Std-Age	−0.120*	−0.171**	−0.163
	(−1.70)	(−2.30)	(−1.50)
Education level(Not receiving education = 0)			
Primary education	−0.885***	−0.882***	−0.538*
	(−4.79)	(−4.78)	(−1.83)
Secondary education	−2.365***	−2.339***	−2.119***
	(−11.38)	(−11.25)	(−6.84)
Higher education	−3.649***	−3.607***	−3.947***
	(−11.57)	(−11.44)	(−8.58)
Marital status(no spouse = 0)	−1.153***	−1.137***	−0.954***
	(−5.83)	(−5.74)	(−2.85)
Living arrangements(not living with children = 0)	−0.401***	−0.355**	−0.512**
	(−2.76)	(−2.42)	(−2.40)
Financial support from son to father(no financial support = 0)	0.291**	0.307**	0.458**
	(2.04)	(2.15)	(2.12)
Financial support from father to son(no financial support = 0)	−0.878***	−0.867***	−0.709***
	(−5.97)	(−5.89)	(−3.20)
Satisfaction with relationship with children(Unsatisfied = 0)	−4.559***	−4.564***	−5.086***
	(−11.76)	(−11.76)	(−8.82)
Health satisfaction(Unsatisfied = 0)	−5.361***	−5.354***	−5.623***
	(−31.30)	(−31.25)	(−21.29)
Constant term	19.835***	19.928***	19.532***
	(45.75)	(45.66)	(28.22)
Observations	7,591	7,591	3,131
Adjusted R-squared	0.228	0.228	0.249
Prob>F	0.000	0.000	0.000

Data source: China Health and Retirement Longitudinal Study Wave 4 (2018). Standard errors in parentheses, *p < 0.1; **p < 0.05; ***p < 0.01; this table applies mixed OLS regressions.

### 3.3 Robustness test

To examine the connection between intergenerational care and older people’s depressive status, we considered factors such as gender, self-care ability, financial standing, and social insurance status, all of which play a role in the provision of intergenerational care (49, 50). However, the complex relationship between these factors and depressive status can make it difficult to isolate the ‘pure’ relationship between the two. This study aims to address this issue by using multiple regressions, but recognizes that it may be challenging to ensure a linear relationship between control variables and the depressive status of older people. Moreover, it is possible that intergenerational care and depressive status may be inherently related, meaning that individuals with lower levels of depression may be more likely to provide care to others. To control for this, we employ the propensity score matching technique proposed by Rosenbaum and Rubin (51) as a robustness check. We first identify the factors influencing the provision of intergenerational care for older people. Next, we calculate propensity scores using the probit model. Finally, we match the sample based on these propensity scores, ensuring that the characteristics of older people who participate in intergenerational care are as similar as possible to those who do not participate. We use kernel matching and radius matching methods to ensure the reliability of our results.

#### 3.3.1 Balance test

Post-matching, a balance test confirms the optimal balancing of intergenerational care participation across both groups through its standardized deviations. As shown in Table 5, these values fall below 10 after matching, indicating a substantial decrease. All t-test results uphold the initial null hypothesis of no significance in group disparities, demonstrating the efficacy of the matching process in achieving balance.

**Table 5:** Matching variables balance test.

Handling variables	Variable	Mean			% bias	% reduce bias	*t*-test	
		Sample	Treated	Control			*t*	*p* > *t*
Intergenerational care	Gender	U	0.527	0.501	5.2		2.24	0.025
		M	0.527	0.529	−0.5	90.6	−0.20	0.842
	Std-Age	U	−0.370	0.290	−71.1		−30.19	0.000
		M	−0.370	−0.363	−0.7	99.0	−0.35	0.723
	Education level	U	1.155	0.991	20.2		8.76	0.000
		M	1.155	1.130	3.1	84.9	1.26	0.208
	Marital status	U	0.877	0.785	24.9		10.63	0.000
		M	0.877	0.874	0.9	96.4	0.41	0.680
	Living arrangements	U	0.405	0.278	27.1		11.76	0.000
		M	0.405	0.400	1.1	95.9	0.43	0.666
	Financial support from son to father	U	0.628	0.639	−2.2		−0.96	0.337
		M	0.628	0.632	−0.9	59.0	−0.37	0.711
	Financial support from father to son	U	0.323	0.295	5.9		2.56	0.010
		M	0.323	0.321	0.5	92.3	0.18	0.854
	Satisfaction with relationship with children	U	0.957	0.954	1.3		0.57	0.570
		M	0.957	0.957	−0.2	87.1	−0.07	0.944
	Health satisfaction	U	0.746	0.726	4.6		2.00	0.046
		M	0.746	0.743	0.6	86.0	0.27	0.789

Data source: China Health and Retirement Longitudinal Study Wave 4 (2018). This table applies nuclear matching and *t*-test.

#### 3.3.2 Regression based on PSM matched data

In Table 6, our OLS model demonstrates a significant—and robust—negative correlation between intergenerational care and older adults’ depression levels. This result was observed both in the Kernel matching analysis and the Radius matching analysis.

**Table 6:** Intergenerational care and depression of older people based on PSM matched data.

Variables	Nuclear matching	Radius matching
Intergenerational care(no = 0)	−0.348**	−0.345**
	(−2.31)	(−2.29)
Control variables	Yes	Yes
Observations	7,591	7,591

Data source: China Health and Retirement Longitudinal Study Wave 4 (2018). Standard errors in parentheses, *p < 0.1; **p < 0.05; ***p < 0.01; this table applies mixed OLS regressions.

### 3.4 Heterogeneity analysis

#### 3.4.1 Gender heterogeneity analysis

To scrutinize potential gendered disparities regarding the connection between intergenerational care and depression of older adults, the sample was partitioned into male and female subsets. Notably, evidence strongly supported the negative correlation between caregiving and depression among female seniors (−0.586; *p* < 0.01). By contrast, such association proved non-significant for male seniors.

#### 3.4.2 Age heterogeneity analysis

We employed a sample divided into two sub-samples based on age, specifically targeting lower-aged older adults (60–75 years old) and higher-aged older adults (75 years old and above). This enabled us to analyze the possible age differences in the relationship between intergenerational care and the depressive status of older people. The results indicated that there is a significant negative relationship between intergenerational care and the depressive status of lower-aged older adults (60–75 years old), with a regression coefficient of −0.275 (*p* < 0.1). In contrast, the relationship between intergenerational care and the depressive status of higher-aged older adults (75 years old and above) was not found to be significant.

From Table 7, it is evident that there are diverse relationships between intergenerational care and the depression of older people, with these relationships varying according to gender and age. This provides support for hypothesis H2.

**Table 7:** Heterogeneity analysis of intergenerational care status on older people’s depression.

Classification		Depression		
		Coefficient	T	Sample size
Gender	Female	−0.586***	−2.64	3,701
	Male	−0.102	−0.53	3,890
Age	60–75 years	−0.275*	−1.82	6,334
	75+ years	−0.068	−0.14	1,257

Data source: China Health and Retirement Longitudinal Study Wave 4 (2018). Standard errors in parentheses, *p < 0.1; **p < 0.05; ***p < 0.01; this table applies mixed OLS regressions.

### 3.5 Analysis of correlation mechanisms

The previous section empirically examined the relationships between intergenerational care and depression of older adults, while the current section aims to delve into the mechanisms behind these relationships.

#### 3.5.1 Mediating effect

To examine whether social activity mediates the association between intergenerational care and older individuals’ depression, this paper utilized commonly utilized operational recommendations to identify the relationship between intergenerational care and social activity through multiple linear regression, and then further explored the theoretical underpinnings of the connection between social activity and depressive states in older adults. Model 1 in Table 8 indicates that the provision of intergenerational care significantly escalated the level of social activity in older individuals. According to activity theory, mental health in older people positively correlates with social activity (52). Activity theory suggests that “participation in social activities” plays a pivotal role in the lives of older individuals, and independent engagement in interpersonal activities can efficiently prevent the adverse effects of life-altering events such as aging, physical decline, and changes in social status, and afford older individuals more energy to live a positive and active social life (53). Continuing to undertake the social responsibilities and roles in their earlier life stages can aid them in managing negative psychological emotions (54). Therefore, intergenerational care significantly reduced depression and enhanced psychological well-being of older individuals by augmenting their social activities, and social activities acted as a mediating pathway for the relationship between intergenerational care and the depressive status of older adults, thus supporting hypothesis H3.

**Table 8:** Mediating effect of social activities and moderating effect of health satisfaction.

	(1)	(2)
Variables	Social activities	Depression
Intergenerational care(no = 0)	0.044***	−0.334**
	(3.63)	(−2.28)
Intergenerational care*health satisfaction		−0.587*
		(−1.75)
Gender(female = 0)	−0.079***	−1.040***
	(−6.49)	(−7.03)
Std-Age	−0.024***	−0.175**
	(−3.76)	(−2.34)
Education level(Not receiving education = 0)		
Primary education	0.099***	−0.875***
	(6.85)	(−4.74)
Secondary education	0.179***	−2.330***
	(10.34)	(−11.22)
Higher education	0.325***	−3.602***
	(11.53)	(−11.42)
Marital status(no spouse = 0)	−0.065***	−1.141***
	(−4.11)	(−5.76)
Living arrangements(not living with children = 0)	−0.043***	−0.356**
	(−3.45)	(−2.43)
Financial support from son to father(no financial support = 0)	0.028**	0.309**
	(2.39)	(2.16)
Financial support from father to son(no financial support = 0)	0.074***	−0.868***
	(5.91)	(−5.89)
Satisfaction with relationship with children(Unsatisfied = 0)	0.009	−4.559***
	(0.32)	(−11.75)
Health satisfaction(Unsatisfied = 0)	0.053***	−5.362***
	(4.13)	(−31.34)
Constant	0.406***	19.931***
	(12.92)	(45.66)
Observations	7,591	7,591
Adjusted R-squared	0.043	0.229
Prob>F	0.000	0.000

Data source: China Health and Retirement Longitudinal Study Wave 4 (2018). Standard errors in parentheses, *p < 0.1; **p < 0.05; ***p < 0.01; this table applies mixed OLS regressions.

#### 3.5.2 Moderating effect

In order to investigate the impact of health satisfaction on the relationship between intergenerational care and depression in older adults, the current study estimated the interaction coefficients between health satisfaction and intergenerational care. The findings from Table 8 indicate that Model 2 revealed significant negative interaction terms when considering the two indicators of intergenerational care and health satisfaction combined. This finding suggests that health satisfaction possesses a positive moderating effect on the relationship between intergenerational care and depression. As such, it was found that the higher the level of health satisfaction, the more effective intergenerational care was in reducing depression symptoms. Individuals who reported higher levels of health satisfaction were more likely to utilize intergenerational care as a method of valuing their family ties and resources, thus enhancing the relationship between intergenerational care and depressive status. Conversely, those with lower levels of health satisfaction were more likely to perceive intergenerational care as a drain on energy, thus reducing the relationship between intergenerational care and depressive status. Overall, these results provide support for Hypothesis 4, which proposes that health satisfaction plays a critical role in modifying the relationship between intergenerational care and depression in older adults.

## 4 Discussion

This paper shows that caring for grandchildren is prevalent among older individuals in China. It highlights that more attention should be paid to the practice of intergenerational care, which not only brings families closer together but also promotes active aging (4). Furthermore, with the introduction of the three-child policy in China and the increase in women’s employment rates, older individuals caring for their grandchildren has become an effective approach to bridge the childcare gap. Notably, intergenerational care not only taps the human resources of older individuals but also presents significant opportunities for their participation in the socio-economic and family development.

Furthermore, intergenerational care can be more than just “altruistic.” Through the involvement of older people in child care, it alleviates a great deal of the burden on their children. In return, children may provide additional financial, daily, and emotional support to their parents. This financial support can alleviate the financial stress that older people may face due to reduced income post-retirement. Daily care can address the basic difficulties that older people may encounter, particularly in cases of illness, providing them with a sense of security. Emotional support can directly alleviate the loneliness that older people may feel, providing them with positive emotional values through interpersonal interaction and emotional communication. As a result, their mental health can improve, as their stress and depression are relieved.

Nevertheless, it is crucial to remember that intergenerational care should be a moderate practice. When the frequency and duration of intergenerational care are well-balanced, it not only can allow older people to take pleasure in caring for their grandchildren, but also prevent them from feeling drained.

In China, due to the traditional gender roles, women are often seen as the primary caregivers in their family, and hence older women are most commonly assigned the responsibility of intergenerational care. In most instances, grandchildren tend to have a closer bond with their grandmothers than with their grandfathers (55). This fondness for their grandchildren further encourages female older adults to become more involved in intergenerational care. As the main caregivers, older women derive joy and personal worth from intergenerational care (56), which in turn, can effectively alleviate depression. However, the connection between intergenerational care and depression is not as significant for grandfathers. For instance, it may be more beneficial for grandfathers to focus their energy on other aspects, such as managing household affairs. Through effective division of labor, the grandparents can alleviate their own stress and provide support for each other, thus promoting their overall mental health.

For younger older individuals, engaging in intergenerational care can provide them with a sense of fulfillment, and can occupy a significant portion of their lives. There is a strong correlation between intergenerational care and their psychological well-being, and appropriate frequency and duration of intergenerational care can provide them with a sense of satisfaction. However, for higher-aged older adults, who may not have the physical capabilities to care for their grandchildren, shifting this responsibility can be overwhelming. Considering the age difference and the physical condition of these individuals, it is important to avoid providing intergenerational care or reduce their care time.

This article examines the mechanisms of intergenerational care and its impact on older people’s depressive status. Social activities play an intermediary role in the relationship between intergenerational care and older people’s depression. Participation in social activities not only maintains social networks, but also enables older people to engage in community exchanges by providing care to their grandchildren. These social interactions can bring them closer to their neighbors, friends, family, and broaden their social and life circles, thereby reducing the likelihood of social isolation. Additionally, a positive relationship exists between health satisfaction and intergenerational care for older people. For those with high levels of health satisfaction, intergenerational care can serve as a pathway to personal fulfillment and the positive effect on their mental health becomes more evident.

Intergenerational care is not unique to China. Professor Buchanan, a representative from Oxford University’s Department of Social Policy, mentioned in the introduction to the 2018 special issue titled “Twenty first century grandparents” that grandparents globally have assumed a more significant role in raising and educating their grandchildren in the last decade (57). Additionally, based on the data from the Australian Bureau of Statistics Time Use Survey of 2006, Craig and Jenkins found that approximately 60% of Australian grandparents surveyed indicated their involvement in caring for their grandchildren (58). In the United States, Guzman reported based on data from two large national surveys (NSFH and NHES) from 1992 to 1994 that 47% of American grandparents were involved in intergenerational care, a significant increase from before the 1990s, as evident by a surge in the number of grandparents living with their grandchildren (59). The differences in intergenerational care among countries around the world are primarily due to specific institutional conditions, cultural rules, and family circumstances. However, the research based on Chinese data on the relationship between intergenerational care and depression in older adults provides insights for promoting the mental health of older people. It was found that promoting intergenerational care for older people can help alleviate depression, and social activities can play a positive role in facilitating this care. Gender and age differences should be considered when providing intergenerational care.

## 5 Conclusion

The significance of intergenerational care for older people should be properly understood by society, families, and individuals, and multiple social actors should be fully mobilized to maximize the benefits of intergenerational care. This includes promoting the involvement of grandparents in caring for their grandchildren, the government’s support for such intergenerational care, the promotion of community activities that offer opportunities for intergenerational interaction, the improvement of mental health services for older people providing intergenerational care, and the moderation of intergenerational care duration and frequency. In doing so, we can promote the concept of active aging, which acknowledges the potential productivity and happiness of older people.

## 6 Limitations

This research study possesses several limitations that should be acknowledged. Firstly, due to constraints related to time and sample loss, the data used in this study were collected from one period. However, as survey questionnaires have time constraints in tracking information, it is possible that some previous caregivers were missed. Furthermore, social desirability and recall bias may result in measurement errors. Secondly, the study solely examined the relationship between intergenerational care and depression in older individuals, without concluding a causal link. Depression is a complex phenomenon, which can be affected by numerous conditions; this study has merely focused on one. Thirdly, the participants in our interviews were from China, and caution is advised when attempting to generalize the results to other countries.

## Data availability statement

Publicly available datasets were analyzed in this study. This data can be found here: http://charls.pku.edu.cn/.

## Author contributions

SH: Data curation, Investigation, Supervision, Writing – original draft. JG: Formal analysis, Investigation, Visualization, Writing – original draft. JX: Methodology, Writing – review & editing.
